# Preoperative Physical Activity Level and Exercise Prescription in Adults With Obesity: The Effect on Post-Bariatric Surgery Outcomes

**DOI:** 10.3389/fphys.2022.869998

**Published:** 2022-07-06

**Authors:** Georges Jabbour, Rony Ibrahim, Nicola Bragazzi

**Affiliations:** ^1^ Physical Education Department, College of Education, Qatar University, Doha, Qatar; ^2^ Laboratory for Industrial and Applied Mathematics (LIAM), Department of Mathematics and Statistics, York University, Toronto, ON, Canada; ^3^ Department of Health Sciences (DISSAL), Postgraduate School of Public Health, University of Genoa, Genoa, Italy

**Keywords:** bariatric surgery, physical activity, exercise intervention, pre-operative, health outcomes

## Abstract

This systematic review summarizes current evidence on the relation between preoperative physical activity (PA) levels with bariatric surgery (BS) outcomes and on the beneficial role of preoperative exercise/PA program among BS candidates. This systematic review suggests that candidate patients accumulating the preoperative PA level improved several BS outcomes. These improvements were reported mainly for anthropometric and cardiometabolic parameters and physical function. Observed improvements manifested during a distinct period of time in response to a wide variety of exercise programs. Evidence on the preoperative PA level as well as on preoperative exercise implementation on BS outcomes is advocated despite the small number of participants and lack of control. Thus, further studies are required to explore the most effective and suitable form of exercise prescription prior to BS while considering physical and psychological limitations of obese patients.

## Introduction

Since severe obesity is associated with several health, physical, and psychological impairments (WHO Consultation on Obesity, 2000; Flegal et al., 2007; Engin, 2017; Pan et al., 2017), bariatric surgery (BS) is widely accepted as a valuable strategy to improve these alterations (Jabbour and Salman, 2021) and related comorbidities (Brethauer et al., 2011; Schauer et al., 2012; Li et al., 2014; Ardestani et al., 2015; Sams et al., 2016) in both short and long term. Despite all of these promising attributes, the large intersubject variabilities in the number of intra- and postoperative complications, as well as the length of operating time and hospital stay, remain unexplained (Fernandez Jr et al., 2004; Steinbrook, 2004; Nguyen et al., 2013).

While the exact reason for this large intersubject variability of BS outcomes is unclear, it seems that a greater preoperative fitness level and an elevated insulin sensitivity are linked to better post-BS outcomes (Gilbertson et al., 2017), while a lower cardiorespiratory fitness (i.e., VO2max < 15.8 ml/kg/min) is associated with a longer operating time, intubation duration, estimated blood loss during surgery, and more frequent cardiovascular complications (Gilbertson et al., 2020).

In their pilot trial, Gilberston et al. (2020) reported that prescribing aerobic exercise at the preoperative stage in addition to standard medical care induced significant improvements in postoperative BS outcomes when compared to standard medical care alone. According to these authors, these improvements may be mediated by fitness-related adaptations, including a reduction in adipose tissue-derived hormones, preservation of lean mass, and enhanced metabolic flexibility. Additional studies are still necessary to better profile the potential benefit of adding aerobic exercise and/or other forms of exercise to improve health parameters in bariatric patients. Thus, enhancing fitness indicators and metabolic parameters, prior to surgery, may improve patient outcomes (McCullough et al., 2006; Gilbertson et al., 2017).

The purpose of the present systematic review was to review the available evidence for the beneficial health impact of adding exercise to SC preoperatively and to address metabolic health and surgical outcomes compared to SC alone in patients receiving BS. Moreover, this systematic review highlights the exercise form and modality being implemented in obese patients.

## Materials and Methods

### Eligibility Criteria

This systematic review was conducted in accordance with the Preferred Reporting Items for Systematic reviews and Meta-Analyses (PRISMA) statement (Moher et al., 2009). The Population, Intervention, Comparator, Outcomes, and Study design (PICOS) approach was used to identify the inclusion criteria (Table 1). Studies that have examined the effects of implementing a structured exercise or a physical activity intervention before BS on postoperative outcomes “body composition, weight loss, physical activity level, performance, and metabolic parameters” were eligible for inclusion. The studies were included in the current systematic review if they met the following criteria: 1) published in peer-reviewed journals, 2) included adult participants, and 3) compared BS outcomes pre- and/or postoperatively. The studies were excluded if they 1) reported only subjective measures, 2) were not written in English, or 3) were retrospective. Moreover, review articles were not included in the current systematic review.

**TABLE 1:** PICOS criteria for inclusion of studies.

Parameter	Inclusion criteria
Population	Bariatric surgery candidates
Intervention	Preoperative physical activity and/or exercise intervention
Comparator	Preoperative vs. postoperative
Outcomes	Fitness level, body weight and composition, physical activity level, physical functioning, and muscular performance, aerobic fitness, metabolic parameters, and hospital stay
Study design	Randomized control trial, intervention trial, and prospective studies

### Literature Search Strategy

Literature searches were conducted in four electronic databases, including PubMed, Institute for Scientific Information (ISI) Web of Knowledge, Web of Science, and SPORTDiscus, to identify studies of preoperative exercise intervention or preoperative PA practices using the search terms “bariatric surgery” or “weight loss surgery” or “obesity surgery” or “weight reduction surgery” or “biliopancreatic diversion” or “laparoscopic band” or “lap and” or “gastric band” or “gastric bypass” or “gastroplasty” or “gastric sleeve” or “sleeve gastrectomy” and “preoperative exercise intervention” or “preoperative physical activity” or “preoperative lifestyle modification.”

The search was completed with a manual search of reference lists of key articles. Since the scope of this review is large in terms of outcome measures, a systematic review and not a meta-analysis was performed.

### Study Selection

The final screening was performed by the principal investigator (GJ) based on the relevance of the inclusion and exclusion criteria and the identified items for assessing the effects of preoperative exercise intervention on anthropometric characteristics and body composition (e.g., body mass, body fat, and BMI), physical performances (e.g., muscular strength and physical capacity), cardiorespiratory fitness and function (e.g., oxygen uptake and heart rate), energy expenditure and metabolism parameters (e.g., resting metabolic rate, insulin resistance, and lipid profile), and hospital stay in obese adults of both genders undergoing BS using PICOS criteria. If the citation showed any potential relevance, the abstract was screened. When abstracts indicated potential inclusion, full-text articles were reviewed.

## Results

### Study Selection and Description

**FIGURE 1:**
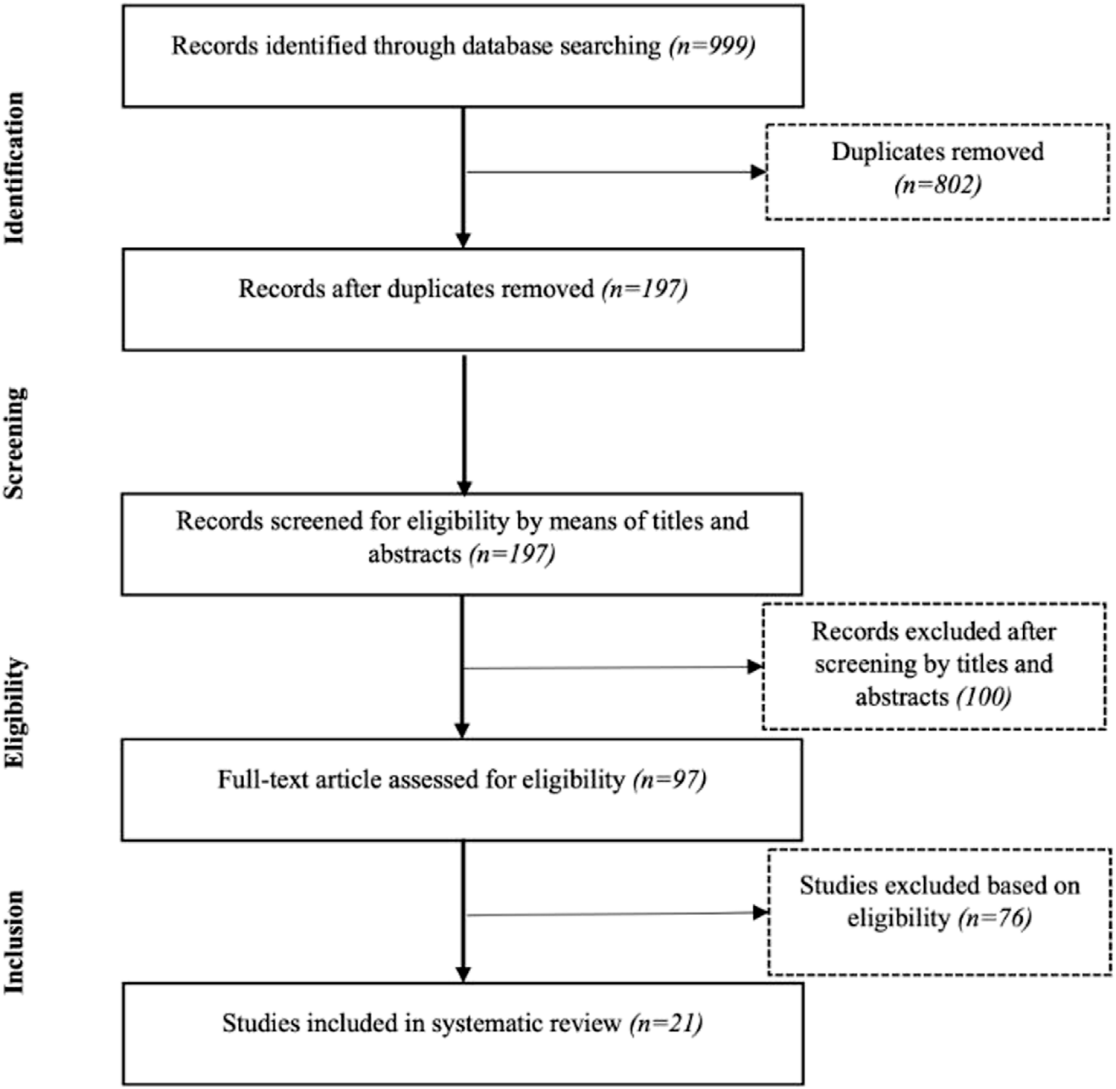
Flow diagram of included and excluded studies included in this systematic review using the recommendations in the Preferred Reporting Items for Systematic reviews and Meta-Analyses (PRISMA) statement.

Our primary research identified 999 records, including 802 duplicates (Figure 1). After screening titles, abstracts, and full texts, 21 studies were included in our final analysis, and the characteristics of these studies are displayed in Table 2.

**TABLE 2:** Baseline characteristics of studies included in the review.

Author (year)	Study design	Population		Baseline BMI (SD)	Type of intervention	Type of bariatric surgery	Assessment methods	Assessment period	Main outcomes
		Age in years; mean (SD) or median [25–75 percentile]	Gender	BMI in kg/m^2^; mean (SD) or median [25–75 percentile]					
Baillot et al. (2013)	RCT	40.8 [37.6–47.5]	Eight F and four M	51.4 [43.8–53.1]	Endurance and strength training	Not specified	Bioimpedance scale, symptom-limited cardiac exercise test, 6-min walk test, sit-to-stand test, half-squat test, and arm curl test	Pre- and 12 weeks post-training	Anthropometric measures and physical fitness
(Baillot et al. (2016)	RCT	43.2 (9.2)	GC (11 F and four M) and GI (12 F and three M)	47.5 (8.1)	Endurance and strength training	Not specified	Bioimpedance scale; symptom-limited exercise test; 6-min walk test; sit-to-stand test; half-squat test; arm curl test; PEBQ; and International PA Questionnaire-Short Form	Pre- and 12 weeks post-training	Anthropometric measures; physical fitness; and physical activity level
Baillot et al. (2017)	RCT	GI [44.8 (39.6–54.7)]; GI2 [45.1 (38.6–55.1)]; and GC [43.5 (37.0–46.2)]	Six F	GI [46.6 (39.2–48.5)]; GI2 [44.4 (40.7–53.5)]; and GC [48.4 (40.6–53.3)]	Endurance and strength training	Not specified	Bioimpedance scale; symptom-limited cardiac exercise test; 6-min walk test; sit-to-stand test; half-squat test; arm curl test; and PEBQ	Pre- and 12 weeks post-training	Anthropometric measures; physical fitness; exercise beliefs; and telehealth perception
Baillot et al. (2018)	RCT	GI [44.5 (8.8)] and GC [41.1 (10.3)]	GI (11 F and two M) and GC (nine F and three M)	Not reported in the text	Endurance and strength training	Roux-en-Y gastric bypass or sleeve gastrectomy	Physical activity intensity and total daily energy expenditure; bioimpedance scale; symptom-limited cardiac exercise test; 6-min walk test; sit-to-stand test, half-squat test; arm curl test; and PEBQ	Pre- and 12 weeks post-intervention and 2 weeks pre- and, 3, 6, 9, and 12 months post-BS	Number of steps; PA intensity; physical fitness; exercise beliefs; and anthropometric measures
Bond et al. (2015b)	RCT	GI [44.2 (9.2)] and GC [48.1 (8.1)]	GI (34 F and six M) and GC (31 F and four M)	GI (45.6 (7.0) and GC (44.4 (5.8))	PA intervention	Not specified	PA (SenseWear Armband) and body mass	Pre- and 6 weeks post-intervention	Daily bout-related moderate-to-vigorous PA and body mass
Bond et al. (2015a)	RCT	GI [44.2 (9.2)] and GC [48.1 (8.1)]	GI (34 F and six M) and GC (31 F and four M)	GI [45.6 (7.0)] and GC [44.4 (5.8)]	PA intervention	Not specified	PA (SenseWear Armband); demographic questionnaire; and body mass	Pre- and 6 weeks post-intervention	Moderate-to-vigorous PA and number of steps per day
Bond et al. (2017)	RCT	GI [46.4 (9.1)] and GC [47.9 (6.8)]	GI (20 F and two M) and GC (11 F and three M)	GI [46.7 (7.1)] and GC [44.4 (7.1)]	PA intervention	Not specified	PA (SenseWear Armband)	Pre-, post-intervention (6 weeks), and post-BS (6 months)	Moderate-to-vigorous PA and number of steps per day
Daniels et al. (2018)	RCT	44.9 (10.2)	16 F	Not reported	Resistance training	Roux-en-Y gastric bypass surgery	Air displacement plethysmography (BodPod); magnetic resonance imaging; and 1-repetition maximum	Pre- and 12 weeks post-training	Fat-free mass; muscle cross-sectional area; muscular strength; and muscle quality
Funderburk and Callis, (2010)	RCT	GI (37.25) and GC (49.3)	Six F (three in each group)	Not reported	Aquatic exercise	Gastric by-pass surgery	Short-Form Health Survey version 2; Obesity Adjustment Scale; Beck Depression Inventory; specialized weight scale; and 6-min walk test	Pre- and post-training	Psychosocial status; depression; adjustment to obesity; and physical status
Gilbertson et al. (2020)	IT	GI [45.6 (4.8)] and GC [39 (5.3)]	GI (seven F) and GC (six F and one M)	GI [43.9 (4.2)] and GC [46.4 (3.0)]	Aerobic exercise	Roux-en-Y gastric bypass Or sleeve gastrectomy	Matsuda index; indirect calorimetry; VO_2_ peak; air displacement plethysmography (BodPod); and mixed meal tolerance test	Pre- and post-training	Insulin sensitivity; metabolic flexibility; aerobic fitness; body composition; and adipokines level
Marcon et al., (2011)	IT	42.5 (12.5)	Seven M and 23 F	48.3 (7.2)	Aerobic exercise	Not specified	Body mass; 6-min walking test; blood pressure; and Framingham score risk	Pre- and post-training	Body mass; BMI; functional capacity; and cardiometabolic risk
Marcon et al. (2017)	RCT	GI [43.4 (2.3)]; GI2 [50.1 (2.8)]; and GC [42.5 (2.7)]	GI (18 F and four M); GI2 (17 F); and GC (16 F and two M)	GI [50.8 (9.6)]; GI2 [45 (4.1)]; and GC [47.1 (7.6)]	Low-intensity exercise program	Not specified	Anthropometric; 6-min walking test; resting heart rate; post-exercise heart rate; pre- and post-exercise respiratory rate; oxygen saturation; and estimated VO_2_ peak	Pre- and post-training	Body mass; BMI; functional capacity; and cardiometabolic risk
Alger-Mayer et al. (2008)	LS	45.3 (8.9)	120 F and 30 M	52.2 (9.8)	No intervention	Gastric bypass surgery	Body mass	Pre- and 3 years and 4 years post-BS	Body mass and BMI
Hickey et al. (1999)	IT	Not found	Not found	Not found	Endurance training	Not specified	Blood test	Not found	Fasting plasma insulin and glucose and lipid concentration
King et al. (2008)	OS	44.6 (11.2)	153 M and 604 F	47.4 (7.6)	No intervention	Not specified	PA (the StepWatch 3 Activity Monitor)	Pre-BS	Steps/day
King et al. (2012)	LS	46 [37–55]	241 F and 69 M	45.4 [41.7–51.2]	No intervention	Not specified	PA (the StepWatch 3 Activity Monitor)	Pre- and 1-year post-BS	Steps/day
Still et al. (2007)	LS	45 Ardestani et al. (2015)	692 F and 192 M	51.3 Li et al. (2014)	Medical, psychological, nutritional, and surgical interventions and education	Roux-en-Y gastric bypass	Anthropometric measures	Pre- and 1-year post-BS	Body mass
Colles et al. (2008)	OS	45.2 (11.5)	103 F and 26 M	44.3 (6.8)	No intervention	Laparoscopic adjustable gastric banding	Anthropometric measures; the Cancer Council Victoria Food Frequency Questionnaire; the Three-Factor Eating Questionnaire; the Beck Depression Inventory; the Physical Component Summary; and the Baecke Physical Activity Questionnaire	Pre- and 4 and 12 months post-BS	Body mass; BMI; total energy expenditure; body composition; TFEQ score; the Beck Depression Inventory score; the Physical Component Summary score; and the Baecke PA scores
Browning et al. (2014)	OS	43.3 Nguyen et al. (2013)	145 F and 27 M	43.8 (5.1)	No intervention	Laparoscopic adjustable gastric banding	Anthropometric measures; presence of comorbidities; and the International Physical Activity Questionnaire (IPAQ)	Pre- and 3, 6, and 12 months post-BS	Body mass; BMI; and PA intensity
Brandenburg and Kotlowski (2005)	IT	46.3 [28–64]	55 F and 15 M	55.3 [36–88]	Behavior modification program	Roux-en-Y gastric bypass	Anthropometric measures and Bariatric surgery questionnaire	1 year post-BS	Body mass; BMI; patient demographics; health information; lifestyle habits; and program information
García-Delgado et al. (2021)	RCT	40 Ardestani et al. (2015)	14 F and one M	46.7 (5.9)	Control group: therapeutical, educational, and cognitive-behavioral therapy; intervention group: therapeutical, educational, and cognitive-behavioral therapy + hysical conditioning and respiratory muscle training program	Not specified	Anthropometric measures; clinical history, physical examination and basic blood tests; EuroQol-5D–5L questionnaire, MEDAS, Eating Disorder Inventory, and Hospital Anxiety and Depression Scale; 6-min walking test, handgrip strength, pulmonary function, and obstructive sleep apnea	Pre-intervention, post-intervention, and post-BS	Body mass; body composition; comorbidities; changes in eating behaviors; health-related quality of life; functional capacity; length of hospital stay after surgery; and short-term complications of surgery

RCT, randomized controlled study; F, female; M, male; GC, group control; GI, group with intervention, LS, longitudinal study; OS; observational study; TFEQ, the Three Factor Eating Questionnaire; PEBQ, physical exercise belief questionnaire; PA, physical activity; BS, bariatric surgery; IT, intervention trial; BMI, body mass index; MEDAS, Mediterranean Diet Adherence Screener questionnaire.

Out of 21 studies, 19 were prospective cohorts (Hickey et al., 1999; Still et al., 2007; Alger-Mayer et al., 2008; Colles et al., 2008; Funderburk and Callis, 2010; Marcon et al., 2011; King et al., 2012; Baillot et al., 2013; Browning et al., 2014; Bond et al., 2015a; Bond et al., 2015b; Baillot et al., 2016; Baillot et al., 2017; Bond et al., 2017; Marcon et al., 2017; Baillot et al., 2018; Daniels et al., 2018; Gilbertson et al., 2020; García-Delgado et al., 2021) and compared pre- to post-BS or pre- to postintervention outcomes in adult patients (Table 2). In total, 14 studies introduced an intervention pre-BS, of which 10 were randomized controlled trials that used a structured exercise program (Funderburk and Callis, 2010; Marcon et al., 2011; Baillot et al., 2013; Baillot et al., 2016; Baillot et al., 2017; Marcon et al., 2017; Baillot et al., 2018; Daniels et al., 2018; Gilbertson et al., 2020; García-Delgado et al., 2021), three studies used a physical activity program (Bond et al., 2015a; Bond et al., 2015b; Bond et al., 2017), and one study used a lifestyle modification program (Still et al., 2007) (Table 2). Among the 14 studies with an intervention, 11 studies performed a pre- to postintervention comparison, nine with exercise (Funderburk and Callis, 2010; Marcon et al., 2011; Baillot et al., 2013; Baillot et al., 2016; Baillot et al., 2017; Marcon et al., 2017; Daniels et al., 2018; Gilbertson et al., 2020), and two with PA intervention (Bond et al., 2015a; Bond et al., 2015b), while the remaining three studies performed a pre- to post-BS comparison (Still et al., 2007; Bond et al., 2017; Baillot et al., 2018) (Table 2).

### Pre- vs. Post-Training Body Composition Changes and Weight Loss

Nine studies reported the effect of exercise training on anthropometric variables, among which five studies found no changes induced by the intervention (Table 3). Baillot et al. (2013) found a reduction in body mass, BMI, and fat mass after 12 weeks of supervised combined endurance and strength training (PreSET). In another study, Baillot et al. (2018) compared BMI between usual care and PreSET groups on seven different occasions (preintervention, 12 weeks postintervention, 2 weeks pre-BS, and 3, 6, 9, and 12 months post-BS). They found that the PreSET group experienced a greater decrease in BMI than the usual care group at 9 and 12 months post-BS. Furthermore, (Marcon et al. (2011 and 2017) reported larger decreases in body mass and BMI in the experimental groups than in the control group in two studies (Table 3). Funderburk and Callis (2010) reported a reduction in body mass after 12 weeks of supervised aquatic exercises, without a difference between the aquatic exercise and control groups. The rest of the studies did not find any anthropometric differences when comparing pre- to postintervention states or when comparing experimental (with exercise intervention) to control groups (without exercise intervention) (Hickey et al., 1999; Baillot et al., 2016; Baillot et al., 2017; Daniels et al., 2018; Gilbertson et al., 2020) (Table 3).

**TABLE 3:** Pre- vs. postoperative body composition, weight loss, physical activity level, performance, and metabolic parameters.

Author (year)	Exercise/PA intervention type	Supervised	Standard medical care and/or outpatient control period	Intervention period	Outcome	Pre-post	Control group
Baillot et al. (2013)	30 min of endurance activity (treadmill and walking circuit) and 20–30 min of strength exercises (upper body, lower body, and trunk)	Yes	Yes	12 weeks	1- body weight (kg)	*	
					2- BMI (kg/m2)	*	
					3- FFM (kg)	*	
					4- FM (kg)	*	
					5- 6MWT (m)	*	
					6- arm curl test (n)	*	
					7- sit-to-stand test (n)	-	
					8- half-squat test (s)	-	
					9- QOL	*	
Baillot et al. (2016)	30 min of endurance activity (treadmill and walking circuit) and 20–30 min of strength exercises (upper body, lower body, and trunk)	Yes	Individual lifestyle counseling intervention	12 weeks	1- body weight (kg)	-	-
					2- fat mass (%)	-	-
					3- SBP (mm Hg)	-	-
					4- DBP (mm Hg)	-	-
					5- 6MWT (m)	-	*
					6- 6MWT perceived exertion	-	-
					7- 6MWT pain (% of subjects)	*	*
					8- 6MWT heart cost	-	*
					9- 6MWT pain intensity scores	*	-
					10- sit-to-stand test (n)	-	-
					11- half-squat test (s)	-	*
					12- arm curl test (n)	-	*
					13- vigorous PA (min/week)	*	*
Baillot et al. (2017)	In-home TelePreSET [supervised twice weekly using videoconferencing] endurance and strength training	Yes	Yes	12 weeks	1- 6MWT distance (m)	*	*
					2- heart cost (m/beats min−1)	*	*
					3- sit-to-stand repetition (n)	*	*
					4- half-squat test time (s)	*	*
					5- arm curl repetition (n)	*	*
					6- maximal aerobic capacity (METS)	*	*
Baillot et al. (2018)	30 min of endurance activity (treadmill and walking circuit) and 20–30 min of strength exercises (upper body, lower body, and trunk)	Yes	Individual lifestyle counseling intervention	12 weeks	1- 1-Y after BS BMI	*	
					2- 1-Y after BS steps (n)	*	
					3- 1-Y after BS light PA (h/day)	*	
					4- 1-Y after BS moderate PA (h/day)	*	
					5- 1-Y after BS 6MWT heart cost	*	
					6- 1-Y after BS half-squat test	*	
Bond et al. (2015b)	Individual face-to-face counseling sessions walking exercise performed at a moderate intensity and in bouts ≥10 min by 30 min/day. A secondary goal was to increase steps taken by 5,000/day	No	Not reported	6 weeks	1- MVPA (minutes/day)	*	*
					2- physical function	-	*
					3- role-physical	*	*
					4- bodily pain	*	*
					5- general health	-	*
					6- mental health	-	-
					7- vitality	-	*
					8- physical component summary	*	*
Bond et al. (2017)	Individual face-to-face counseling sessions walking exercise performed at a moderate intensity and in bouts ≥10 min by 30 min/day. A secondary goal was to increase steps taken by 5,000/day	Yes	Not reported	6 weeks	1–6-month after BS steps per day (n)	*	*
					2–6-months after BS MVPA (minutes/day)	-	-
Daniels et al. (2018)	Period 1, three training sessions per week (8–10 exercises, 1 set per exercise, at a range of 10–15 repetitions per set and an intensity of 50–60% of one-repetition maximum (1-RM); period 2, weeks 2–7, consisted of progressively higher volume workouts (i.e., 8–10 exercises, 3–4 sets, and 10–15 repetitions), and progressively higher resistance/intensity (70–80% 1-RM); and period 3 consisted of the remaining 5 weeks of the 12-week resistance-training program. To increase the resistance/intensity (>80% 1-RM) of the exercises from period 2 and decreasing the number of repetitions to 8–12	Yes		12 weeks	Body weight (kg)	*	-
					Stands for leg press (kg)	*	*
					Stands for leg extension (kg)	*	*
					Stands for quadriceps cross-sectional area (cm^2^)	-	-
					Stands for whole thigh cross-sectional area (cm^2^)	-	-
					Stands for muscle quality leg press	*	*
					Stands for muscle quality leg extension	*	*
Funderburk and Callis (2010)	60 min of aquatic exercises including endurance and strength exercises	Yes	Not reported	12 weeks	1- body weight (kg)	*	-
					2- SBP(mm Hg)	*	-
					3- DBP (mm Hg)	*	-
					4- 6MWT (m)	*	-
					5-RPE	*	-
					5- QOL	*	-
					6- depression score	-	-
					7- physical functioning	*	*
					8- role-physical	*	*
					9- general health	*	*
					10-vitality	*	*
					11- bodily pain	-	-
					12- social functioning	-	-
					13- role-emotional	-	-
					14- mental health	-	-
Gilbertson et al. (2020)	Home basis walking at 65–85% of the HR peak for 30 min per day and 5 days per week	No	Met with dieticians, attended an education session and were cleared for bariatric surgery by a psychologist + for 2 weeks prior to surgery, patients instructed by registered dieticians to consume a meal replacement shake	30 days	1- body weight (kg)	-	-
					2- BMI (kg/m^2^)	-	-
					3- FFM (kg)	*	*
					4-VO2 peak (ml/kg/min)	*	*
					5- glucose (mg/dl)	-	-
					6- FFA (mEq/l)	-	-
					7- insulin (µU/ml)	-	-
					8- adiponectin	*	*
					9- resting metabolic rate (kcal/kgBW/d)	*	-
					8- length of hospital (min)	*	*
Hickey et al. (1999)	Endurance training at 60% of the VO2 peak and each session was 60 min	Yes	A 3-day outpatient control period was used to monitor adequate caloric intake using dietary intake questionnaires	7 days	1- fasting plasma insulin (pmol)	*	
					2- body weight (kg)	-	
					3- % FM	-	
					4- glucose (mg/dl)	-	
					5- lipid (mg/dl)	-	
					6- VO2 peak	-	
Marcon et al. (2011)	Low intensity endurance training; one session per week, consisting of 209 min of exercise and 10 min stretching	Yes	Not reported	24 weeks	1- body weight (kg)	*	
					2- BMI (kg/m^2^)	*	
					3- SBP (mm Hg)	*	
					4- DBP (mm Hg)	*	
					5- TC (mg/dl)	*	
					6- HDL-C (mg/dl)	*	
					7- LDL-C (mg/dl)	*	
					8- TG (mg/dl)	*	
					9- glucose (mg/dl)	*	
					10- 6MWT (m)	*	
Marcon et al. (2017)	Aerobic and stretching exercises performed in two weekly sessions of up to 25 min each, and patients were encouraged to increase the number of steps walked daily for 4 months	Yes	Routine treatment for the control group and support group sessions for lifestyle modification (for EXER + CBT group)	4 months	1- weight (kg)	*	*
					2- BMI (kg/m2)	*	*
					3-heart rate exercise (bpm)	*	*
					4- SBPrest (mmHg)	*	*
					5- SBP post-exer (mmHg)	*	*
					6- DBPrest (mmHg)	*	*
					7- DBP post-exer (mmHg)	*	*
					8- HDL-C (mg/dl)	*	*
					9- TC (mg/dl)	*	*
					10- TG (mg/dl)	*	*
					11- glucose (mg/dl)	*	*
García-Delgado et al. (2021)	15–20 min of physical conditioning consisting of four resistance exercises with elastic bands (2–3 sets of 15 repetitions per exercise) and 10 min of respiratory muscle training consisting of incentive spirometry, respiratory exercises, and inspiratory muscle training.	Yes	Standard medical care for the control group. In addition, the intervention group performs a specific prehabilitation program	16 weeks	1- body mass	Small sample size no. statistics	Small sample size no. statistics
					2- body composition		
					3- comorbidities		
					4- eating behaviors		
					5- health-related quality of life		
					6- functional capacity		
					7- hospital stay post-surgery		
					8- short-term complications of surgery		

6MWT, 6-min walking test; n, number; SBP, systolic blood pressure; DBP, diastolic blood pressure; VO2 peak, maximum oxygen consumption; TC, total cholesterol; TG, triglycerides; FPG, fasting plasma glucose; FPI, fasting plasma. insulin. (*, 0.05; **, 0.01).

### Pre- vs. Post-Training Effects on Physical Fitness Parameters

Nine studies reported the effect of exercise intervention on physical fitness parameters (Funderburk and Callis, 2010; Marcon et al., 2011; Baillot et al., 2013; Baillot et al., 2016; Baillot et al., 2017; Marcon et al., 2017; Baillot et al., 2018; Daniels et al., 2018; Gilbertson et al., 2020), of which two found no changes in measured parameters induced by the intervention (Marcon et al., 2017; Gilbertson et al., 2020) (Table 3). All four studies that used concurrent training (endurance and strength training) reported improvement in cardiovascular and/or muscular fitness parameters (Baillot et al., 2013; Baillot et al., 2016; Baillot et al., 2017; Baillot et al., 2018). Baillot et al. (2013 and 2016) compared patients’ baseline measures to 12 weeks post-training. They found an improvement in the 6-min walk test (6MWT) distance, percentage of theoretical 6MWT distance reached, 6MWT heart cost, half-squat test, and arm curl test. No differences were found in the sit-to-stand test or maximum aerobic capacity. In another study, Baillot et al. (2017) performed within (pre- and 12 weeks post-training) and between groups (training vs. conventional care group) comparisons. Compared to baseline measures, 6MWT distance, sit-to-stand repetitions, arm curl repetitions, and maximal aerobic capacity improved after 12 weeks of training. However, only the 6MWT distance, arm curl repetitions, and 6MWT heart cost improved in the training group compared to the conventional care group (Table 3). Another study between-group comparison revealed an improvement in 6MWT heart cost and the half-squat test for the training compared to the conventional care group (Baillot et al., 2018). Notably, the 6MWT distance was found to improve in two studies after aerobic training programs (Marcon et al., 2011) and aquatic exercise programs (Funderburk and Callis, 2010). Finally, only one study evaluated the effect of a 12-week resistance training program and found improvements in leg press strength, leg extension strength, and leg press muscle quality (Daniels et al., 2018) (Table 3).

### Pre- vs. Postintervention Effects on Postoperative Complications and Hospital Length Stay

Fourteen studies introduced an intervention, among which only four studies reported health-related parameters (Marcon et al., 2011; Baillot et al., 2016; Marcon et al., 2017; Gilbertson et al., 2020) (Table 3). Baillot et al. (2016) reported that BS candidates who were committed to a 12-week exercise intervention were protected from worsening of musculoskeletal pain. Gilbertson et al. (2020) tested the effect of a pre-BS aerobic exercise intervention on insulin sensitivity, metabolic flexibility, adipokines, and length of hospital stay. Marcon et al. (2011) evaluated the effect of an aerobic exercise program on the cardio-metabolic risk of BS candidates. A significant decrease in systolic and diastolic pressure and the Framingham risk score was found after 6 months of the supervised aerobic exercise program (Table 3).

## Discussion

In general, patients awaiting BS have a reduced physical fitness level and impairments in several metabolic variables and body composition before surgery. Although BS results in significant weight loss and body composition changes after surgery, it remains uncertain whether other health outcomes (e.g., fitness, metabolic, and cardiorespiratory parameters) are sufficiently improved and how long the improvements can be maintained. The present systematic review highlights the importance of implementing PA and/or exercise interventions close to the candidate’s date of surgery (Bond et al., 2006; Bond et al., 2015a; Bond et al., 2015b; Bond et al., 2017). Such interventions could procure many health benefits during the preoperative period (e.g., improved fitness level and PA levels) and in postoperative outcomes (e.g., reduced BS-related complications and reduced hospital length of stay) among BS candidates. Therefore, a preoperative PA/exercise intervention could be an ideal approach to maximize the BS benefits and to offer a successful transition toward improving postoperative lifestyle behaviors among BS candidates. Nevertheless, studies with larger cohorts are needed to confirm these results, and a longer follow-up period (>1 year) is required to understand more fully the impact of a preoperative intervention on postoperative outcomes.

### Pre- vs. Post-Training Body Composition Changes and Weight Loss

Six studies examined the effect of PA and/or exercise intervention on body composition parameters pre- vs. postoperatively (Brandenburg and Kotlowski, 2005; Still et al., 2007; Alger-Mayer et al., 2008; Colles et al., 2008; Browning et al., 2014; Baillot et al., 2018), and five studies reported relevant data regarding body composition parameters before and after PA/exercise intervention in preoperative BS candidates (Baillot et al., 2013; Baillot et al., 2016; Baillot et al., 2017; Marcon et al., 2017; Baillot et al., 2018) (Table 4). Some studies showed a significant decrease in preoperative body mass (Still et al., 2007; Alger-Mayer et al., 2008; Bond et al., 2015a) or BMI (King et al., 2008) after an intervention. For these studies, the positive impact procured by a PA intervention prior to BS may be explained primarily by the improvement in PA and physical fitness levels among BS candidates, which is an important step toward improving their overall health parameters. Despite these promising results, it remains difficult to attribute all of these improvements solely to PA considering that many limitations have not been addressed, such as the lack of any control of PA (in the majority of cases, patients were only advised to practice PA) without excluding the interference of BS candidates’ existing conditions (such as BMI and comorbidities) as well as their diet and lifestyle prior to surgery.

**TABLE 4:** Preoperative physical activity and its effects on candidates’ outcomes.

Authors (year)	Physical activity form	Physical activity measure	Evaluation period	Outcome	Result
Alger-Mayer et al. (2008)	Patients given exercise “advice”	None	Pre-BS	Body mass	↓ Pre-BS body mass
Bond et al. (2015b)	Helping patients to adopt behavior change	The SenseWear Armband monitor (SWA; BodyMedia, Inc., Pittsburgh, PA)	Pre and post-BS	Daily moderate-to-vigorous physical activity; daily steps; and body mass	↑ Pre- and post-BS; ↑ pre and post-BS; and ↓ post-BS in comparison to the control group
Colles et al. (2008)	Standard advice regarding recommended postoperative eating behaviors and exercise patterns	Validated self-report and Questionnaire Physical Component Summary score of the Medical Outcomes Trust Short Form-36 (SF-36)	Pre and post-BS	Body mass and Beck Depression Inventory-depression score	↓ 4 and 12 months post-BS and ↓ 12 months post-BS
King et al. (2008)	Habitual PA	Objective evaluation of total PA and peak PA intensity; [StepWatch™ 3 Activity monitor (SAM, OrthoCare Innovations, Washington, D.C.)]	Pre-BS	BMI	↓ BMI
King et al. (2012)	Not reported	The StepWatch™ 3 Activity monitor; mean step/day, active minutes/day, and high-cadence minutes/week	1 year post-BS	PA level	Although gains in PA may be smaller among patients with higher preoperative PA, preoperative PA had the strongest positive association with post-operative PA.
Still et al. (2007)	Patients encouraged to use pedometer and walk “8,000 steps/day”	Not reported	Pre- and 12 months post-BS	Body mass	↓ in comparison to control group; ↓ 12 months post-BS

BS, bariatric surgery; PA, physical activity; BMI, body mass index.

Nonetheless, the effect of PA and exercise interventions on postoperative anthropometric parameters has been considered an interesting topic. Studies using interdisciplinary individual lifestyle counseling and helping BS patients adopt behavioral changes (e.g., to be active) (Still et al., 2007; Colles et al., 2008; Bond et al., 2015b) reported significant decreases in body mass during the post-BS period (Table 4). However, the lack of a control group, the small sample size, and the specificity of the sample may limit the generalizability of these results.

Other studies (Funderburk and Callis, 2010; Baillot et al., 2016; Baillot et al., 2017; Daniels et al., 2018; Gilbertson et al., 2020) applying supervised exercise training during the preoperative period found no significant pre- to postoperative changes in any body composition parameters between the intervention and usual care groups. It seems that BS induces a strong influence on weight loss and therefore can mask any eventual effect of preoperative intervention. In a study by Baillot et al. (2016), participants received lifestyle counseling for an average of 10.4 ± 4.0 months (5.8 ± 1.8 dietician and 5.6 ± 1.8 PA specialist visits) before inclusion in the study. Thus, significant changes might have occurred before inclusion in the surgical treatment option. In contrast, Baillot et al. (2018) reported a larger BMI decrease in the BS group undergoing preoperative exercise intervention (PreSET) compared to that in the usual care group and attributed this improvement to the higher loss of fat-free mass (FFM) in the PreSET group. Moreover, Gilbertson et al. (2020) reported a significant decrease in FFM in participants undergoing preoperative home-based walking for 30 min per day. In addition, Marcon et al. (2017) reported similar results after 4 months of aerobic and stretching exercises. These discrepancies among results might be primarily attributed to the duration and form of intervention. Moreover, the characteristics of patients prior to the PA and exercise intervention must be considered. In fact, many BS candidates encounter remarkable difficulties (e.g., musculoskeletal problems, preoperative fitness level) that might affect their exercise tolerance and adherence, consequently limiting or reducing the PA/exercise intervention benefits. Therefore, more support in selecting an appropriate activity along with a feasible monitoring technique is highly required in such a context.

### Pre- vs. Post-Training Effects on Physical Fitness Parameters

Reduced physical fitness, reported mostly in BS candidates, may affect the BS results. Current evidence supporting the importance of increasing the physical fitness level in BS candidates is not abundant but is promising. In fact, several studies found that a preoperative intervention based on exercise or Pa that aimed at improving physical fitness and performance indicators (e.g., strength, 6MWT distance, and maximal aerobic capacity) among individuals awaiting BS may be an effective strategy to improve the BS candidates’ overall health parameters and their BS outcomes (Funderburk and Callis, 2010; Baillot et al., 2013; Baillot et al., 2016; Baillot et al., 2017; Marcon et al., 2017; Baillot et al., 2018; Daniels et al., 2018). In this regard, Baillot et al. (2013 and 2016), in their randomized controlled trial, reported a significant increase in some physical function parameters assessed with a test battery (6MWT, sit-to-stand, half-squat, and arm curl test) after 12 weeks of supervised exercise training either with or without an individual lifestyle counseling intervention. One study by Gilbertson et al. (2020) reported significant increases in the VO_2_ peak among BS candidates after adding preoperative aerobic exercise to standard medical care. To the best of our knowledge, this is the only study to investigate the effect of a preoperative PA intervention on aerobic performance. The increase in the VO_2_ peak may be associated with a shorter operation time and length of hospital stay and with prevention of muscle loss along with a concomitant increase in the PA level among BS candidates. However, the mechanism underlying these improvements remains to be studied.

The PA level is an interesting parameter that has been evaluated. In a long-term study (1 year after surgery), Baillot et al. (2018) reported that the addition of preoperative supervised exercise training to individual lifestyle counseling improved PA levels and submaximal physical fitness 1 year post-BS. Similarly, King et al. (2012) reported a gain in the PA level 1 year after surgery among patients with higher preoperative PA levels. Other short-term studies (Bond et al., 2015a; Baillot et al., 2016; Bond et al., 2017) reported similar results regarding PA levels in response to preoperative intervention. A small amount of evidence suggests that improvements in PA in response to a preoperative PA/exercise intervention may mainly be attributed to improvements in PA barriers, social interactions, and feelings of embarrassment (Bond et al., 2015a; Baillot et al., 2016). In addition, the impact of the overall improvement in the fitness level following a PA/exercise intervention aimed at improving the physical fitness of preoperative BS candidates cannot be overlooked. However, the small sample size and exclusion criteria applied in the aforementioned studies prevent their generalization to all subjects awaiting BS. Moreover, the study recruitment process was limited to volunteers who were able to frequently visit the facility and were without major functional limitations.

### Pre- vs. Postintervention Effects on Health Parameters, Postoperative Complications, and Length of Hospital Stay

Only four studies have explored a limited number of health parameters (Marcon et al., 2011; Baillot et al., 2016; Marcon et al., 2017; Gilbertson et al., 2020). Baillot et al. (2016) looked at the effect of a 12-week exercise program on musculoskeletal pain and found that BS candidates can be protected from worsening of pain associated with daily life activities. Additional studies are required to confirm the impact of PA on musculoskeletal pain before and after BS. In a study by Gilbertson et al. (2020), patients undergoing preoperative EX + SC prior to bariatric surgery had a shorter length of hospital stay than patients undergoing preoperative SC. Two studies by Marcon et al. (2011 and 2017) reported a significant decrease in systolic and diastolic blood pressure and the Framingham risk score after 6 months of a supervised aerobic exercise program.

To date, the mechanisms responsible for such improvement have not been explored, although current evidence clearly favors a preoperative PA/exercise intervention for facilitating better postoperative outcomes. Few studies have investigated the role of preoperative interventions on BS outcomes. The differences in study design and the lack of randomized controlled trials decrease the evidence level of the results. Moreover, the heterogeneity of activities performed (with or without supervision), the inclusion criteria, and the interference of covariates (e.g., participant characteristics) were not well controlled. Finally, it is important to mention that the included studies were very small and had a short follow-up time, thus making the results less convincing. The data provided by this review did not consider BS procedures (i.e., type of surgery), making the results difficult to interpret.

In conclusion, this review summarizes the benefits of a preoperative PA/exercise intervention among BS candidates and highlights the importance of such strategies as a component of medical therapy. A good understanding of the beneficial effect of improving the preoperative physical condition on postoperative outcomes is highly recommended for future interventional studies to potentiate the beneficial effect of BS among obese candidates. Based on significant evidence, including optimized weight loss post BS, reduced cardiovascular risk, and increases in patients’ regular PA and fitness levels, there is a need to implement a PA/exercise program before BS to promote and optimize BS outcomes. Nevertheless, there is a need for future investigations in this field to determine the most appropriate form(s) of PA/exercise intervention according to the patient profile.

## Abbreviations

BMI, body mass index; BS, bariatric surgery; BW, body weight; C, abdominal circumference; F, female; FFM, fat-free mass; FM, fat mass; GC, group control; GI, group with intervention; HbA1c, glycated hemoglobin; HOMA-IR, homeostatic model assessment for insulin resistance; HR, heart rate; IT, intervention trial; LS, longitudinal study; M, male; OS, observational study; PA, physical activity; PEBQ, physical exercise belief questionnaire; REE, resting energy expenditure; TEE, total energy expenditure; W/H, waist-to-hip ratio; WC, waist circumference; RCT, randomized controlled study; and TFEQ, three-factor eating questionnaire.
